# Research on the water-entry attitude of a submersible aircraft

**DOI:** 10.1186/s40064-016-3614-2

**Published:** 2016-11-08

**Authors:** BaoWei Xu, YongLi Li, JinFu Feng, JunHua Hu, Duo Qi, Jian Yang

**Affiliations:** 1 Flying Instructor Training Base, Air Force Aviation University, Bengbu, 233000 China; 2 Engineering College of Aeronautics and Astronautics, Air Force Engineering University, Xi’an, 710038 China; 3 Engineering University of CAPF, Xi’an, 710086 China

**Keywords:** Submersible aircraft, Dynamic model, Water-entry, Experiment, Overturning, Optimizing search

## Abstract

**Background:**

The water entry of a submersible aircraft, which is transient, highly coupled, and nonlinear, is complicated. After analyzing the mechanics of this process, the change rate of every variable is considered. A dynamic model is build and employed to study vehicle attitude and overturn phenomenon during water entry. Experiments are carried out and a method to organize experiment data is proposed. The accuracy of the method is confirmed by comparing the results of simulation of dynamic model and experiment under the same condition.

**Results:**

Based on the analysis of the experiment and simulation, the initial attack angle and angular velocity largely influence the water entry of vehicle. Simulations of water entry with different initial and angular velocities are completed, followed by an analysis, and the motion law of vehicle is obtained. To solve the problem of vehicle stability and control during water entry, an approach is proposed by which the vehicle sails with a zero attack angle after entering water by controlling the initial angular velocity. With the dynamic model and optimization research algorithm, calculation is performed, and the optimal initial angular velocity of water-entry is obtained.

**Conclusions:**

The outcome of simulations confirms that the effectiveness of the propose approach by which the initial water-entry angular velocity is controlled.

## Background

Recently, researchers began to focus on submersible aircrafts that not only fly but also navigate underwater. For instance, the detailed design objective of submersible aircraft was proposed in Rick and Jonathan (2010), Kathryn (2011). Water entry and water exit are critical stages of submersible aircraft movement. The alternating repeated movement through air–water medium of the vehicle accompanies the force and moment mutation, gas–liquid disturbance, and complicated collision phenomenon. It can adversely affect vehicle control, which seriously affects vehicle stability, and even cause the overturn phenomenon. To prevent the overturn phenomenon and effectively control the water entry of vehicle, the motion law and attitude control during water entry of submersible aircraft are studied in this work.

Reviewing the results of existing research on water entry, they mainly concentrated on calculating the impacting load (Yettou et al. 2006; Nuffel et al. 2014; Alaoui et al. 2015), changing of cavity shape (He et al. 2012a, b; Li et al. 2010), and free surface (Han et al. 2014; Lu et al. 2008; Zhang et al. 2012). The typical methods adopted were numerical simulation and experiments. In He et al. (2012), the volume-of-fluid method coupled with the dynamic mesh method was used to simulate the movement of the vertical water-entry body. Wang and Shi (2012) built the oblique water-entry numerical model of airborne missile to simulate the initial water-entry hydroballistics. Park and Jung (2003) utilized the numerical analysis method to study the impact force and ricochet behavior of high-speed water-entry bodies. In Hu and Liu (2014a, b), numerical simulation and experiment were respectively used to study the characteristics of flat-bottomed body entering water, and the results indicated the impact forces mainly depend on water-entry velocities. In Liang et al. (2014), the wing root pivot joint’s radial load of a submersible airplane, which imitates the locomotion of gannet’s Morus plunge diving, was studied by implementing a test device named Mimic-Gannet. The outcomes revealed the wing load characteristic during the plunge of gannet. May (1975) collected valuable information and experiment data on water entry and systematically analyzed variables that may affect water-entry trajectory. Zhang et al. (2011), Gu et al. (2012) concluded that the head style is crucial to cavities and water-entry stabilization by studying the water entry of objects with different head styles.

Numerical simulation and experiment were the main approaches used in studies on water entry. Most studies focused on fixed work conditions and special phenomenon, neglecting the change of attitude and control during water entry. Nguyen et al. Nguyen (2014) developed an implicit algorithm based on a dual-time pseudo-compressibility method to compute water impact forces on bodies. And in Nguyen et al. (2016), a 6 DOF rigid body motion model and a moving Chimera grid scheme were developed for multiphase flow around moving bodies with application to the water entry problem.

With theoretical analysis methods, a submersible vehicle is taken as the research object in this study. Under the condition of slow velocity, omitting change of free surface, water jet, and cavity, the force mechanism of the object is analyzed to establish a dynamic model of water entry. Finally, the motion law, attitude change, and control of the object during water entry are studied using the dynamic model and optimum searching algorithm.

## Establishment of dynamical model

### Shape model

**Fig. 1:**
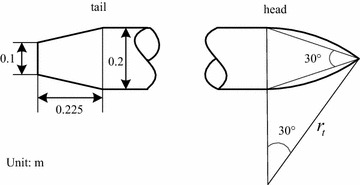
*Contour* of the vehicle (unit: m)

In this study, the peaked arch shape model is used; the model is 2 m long, with a vertex angle of 30° and a uniform mass distribution. It is designed as a linear cutting tail. Figure 1 illustrates the physical model of the vehicle. The computational formula of the radius is shown as the Eq. (1).

1 $$R(x) = \left\{ {\begin{array}{*{20}l} 0 \hfill &\quad {x \le 0\,or\,x > 2} \hfill \\ {0.2222x + 0.05} \hfill &\quad {0 < x \le 0.225} \hfill \\ {0.1} \hfill &\quad {0.225 < x \le 2 - \frac{0.1}{\tan 15^\circ }} \hfill \\ {0.1 + \sqrt {r_{t}^{2} - \left( {x + \frac{0.1}{\tan 15^\circ } - 2} \right)^{2} } - r} \hfill &\quad {2 - \frac{0.1}{\tan 15^\circ } < x \le 2} \hfill \\ \end{array} } \right.$$

In the Eq. (1), $r_{t} = \frac{0.05}{\sin 15^\circ \cdot \sin 15^\circ }$.

### Force analysis

**Fig. 2:**
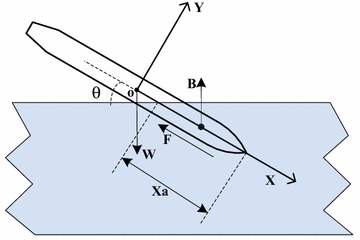
Force analysis of the vehicle crossing water

The body axis coordinate system, which is selected as reference coordinate system, is built with the origin at the center of the mass (Fig. 2). The water-entry angle is *θ*. Considering the minimal effect of air force, the vehicle only interacts with the weight *W*, buoyancy *B*, and fluid force *F* during water entry. The weight *W* remains unchanged, whereas the buoyancy *B* and fluid force *F* change along with *x*_*a*_, which is the water-entry distance of the vehicle.

1. Weight *W*

During the entire water-entry process, the weight *W* remains unchanged.

2 $$\left\{ {\begin{array}{*{20}l} {W = mg} \hfill \\ {m = \rho \cdot V} \hfill \\ {V = \pi \int_{0}^{L} {R^{2} (x)} dx} \hfill \\ \end{array} } \right.$$

In the Eq. (2), *R*(*x*) is the radius of the vehicle, *ρ* is the density and *V* is the volume.

The center-of-gravity position is:

3 $$x_{0} = \frac{{\int_{0}^{L} {\rho \pi R^{2} (x)xdx} }}{m}$$

2. Buoyancy *B*

The magnitude and location of the buoyancy *B* changes along with the water-entry distance *x*_*a*_. The buoyancy *B* is assumed to be unaffected by the variability of the free surface. The buoyancy and location can be calculated as follows:

4 $$B = \rho_{0} g\int_{{L_{in} }}^{{}} {\pi R^{2} (x)dx}$$

5 $$x_{b0} = \frac{{\rho_{0} g\int_{{L_{in} }}^{{}} {\pi R^{2} (x)xdx} }}{B}$$

In Eqs. (4, 5), *ρ*_0_ is the density of water, *L*_*in*_ is the length of the vehicle in the water during water entry, and the data range is [*L* − *x*_*a*_, *L*]. The distance between the center of mass and the center of buoyancy is:

6 $$x_{b} = x_{0} - x_{b0}$$

3. Fluid force *F*

During water entry, the fluid force *F* is difficult to analyze. To analyze the fluid force *F* expediently in this study, it is divided into ideal and viscous fluid for calculation.

➀ Ideal fluid *F*_*i*_

In terms of the theory of momentum and moment of momentum, the vector and moment of ideal force on the vehicle can be obtained as follows:

7 $$- F_{i} = dQ_{f} /dt + \omega \times Q_{f}$$

8 $$- M_{i} = dK_{f} /dt + \omega \times K_{f} + V \times Q_{f}$$

In above equation, *F*_*i*_ is the ideal fluid force on the vehicle, *M*_*i*_ is the ideal fluid torque on vehicle, *Q*_*f*_ and *K*_*f*_ are the ideal fluid force momentum and the moment of momentum on the vehicle, respectively, which can be obtained by the added mass (Yan 2005), *ω* is the rotation angular velocity, and *v* is the velocity vector.

According to the theory of potential flow, *Q*_*f*_ and *K*_*f*_ can be expressed as follows:

9 $$\left[ {\begin{array}{*{20}c} {Q_{fx} } \\ {Q_{fy} } \\ {Q_{fz} } \\ {K_{fx} } \\ {K_{fy} } \\ {K_{fz} } \\ \end{array} } \right] = \left[ {\begin{array}{*{20}c} {\lambda_{11} } &\quad {\lambda_{12} } &\quad {\lambda_{13} } &\quad {\lambda_{14} } &\quad {\lambda_{15} } &\quad {\lambda_{16} } \\ {\lambda_{21} } &\quad {\lambda_{22} } &\quad {\lambda_{23} } &\quad {\lambda_{24} } &\quad {\lambda_{25} } &\quad {\lambda_{26} } \\ {\lambda_{31} } &\quad {\lambda_{32} } &\quad {\lambda_{33} } &\quad {\lambda_{34} } &\quad {\lambda_{35} } &\quad {\lambda_{36} } \\ {\lambda_{41} } &\quad {\lambda_{42} } &\quad {\lambda_{43} } &\quad {\lambda_{44} } &\quad {\lambda_{45} } &\quad {\lambda_{46} } \\ {\lambda_{51} } &\quad {\lambda_{52} } &\quad {\lambda_{53} } &\quad {\lambda_{54} } &\quad {\lambda_{55} } &\quad {\lambda_{56} } \\ {\lambda_{61} } &\quad {\lambda_{62} } &\quad {\lambda_{63} } &\quad {\lambda_{64} } &\quad {\lambda_{65} } &\quad {\lambda_{66} } \\ \end{array} } \right]\left[ {\begin{array}{*{20}c} {v_{x} } \\ {v_{y} } \\ {v_{z} } \\ {\omega_{x} } \\ {\omega_{y} } \\ {\omega_{z} } \\ \end{array} } \right]$$

In above equation, $\lambda_{11} , \lambda_{12} , \ldots ,\lambda_{66}$ is the added mass, which depends only on the shape of the wetted area.

During water entry, the wetted volume of the vehicle and the added mass change along with time (Liao et al. 2012). Therefore, the variation of added mass must be fully considered. In this study, the effect of the changing added mass change and the rate of change are accounted for. Based on Eqs. (7) and (8) and the definition of added mass (Yan 2005), the ideal fluid force equation can be obtained as follows:

10 $$\begin{aligned} \left[ {\begin{array}{*{20}c} {F_{ix} } \\ {F_{iy} } \\ {F_{iz} } \\ {M_{ix} } \\ {M_{iy} } \\ {M_{iz} } \\ \end{array} } \right] & = - {\varvec{\uplambda}}\left[ {\begin{array}{*{20}c} {{\text{d}}v_{x} /{\text{d}}t} \\ {{\text{d}}v_{y} /{\text{d}}t} \\ {{\text{d}}v_{z} /{\text{d}}t} \\ {{\text{d}}\omega_{x} /{\text{d}}t} \\ {{\text{d}}\omega_{y} /{\text{d}}t} \\ {{\text{d}}\omega_{z} /{\text{d}}t} \\ \end{array} } \right] - {\dot{\mathbf{\lambda }}}\left[ {\begin{array}{*{20}c} {v_{x} } \\ {v_{y} } \\ {v_{z} } \\ {\omega_{x} } \\ {\omega_{y} } \\ {\omega_{z} } \\ \end{array} } \right] \\ & \quad - \left[ {\begin{array}{*{20}c} 0 &\quad { - \omega_{z} } &\quad {\omega_{y} } &\quad 0 &\quad 0 &\quad 0 \\ {\omega_{z} } &\quad 0 &\quad { - \omega_{x} } &\quad 0 &\quad 0 &\quad 0 \\ { - \omega_{y} } &\quad {\omega_{x} } &\quad 0 &\quad 0 &\quad 0 &\quad 0 \\ 0 &\quad 0 &\quad 0 &\quad 0 &\quad { - \omega_{z} } &\quad {\omega_{y} } \\ 0 &\quad 0 &\quad 0 &\quad {\omega_{z} } &\quad 0 &\quad { - \omega_{x} } \\ 0 &\quad 0 &\quad 0 &\quad { - \omega_{y} } &\quad {\omega_{x} } &\quad 0 \\ \end{array} } \right]\left\{ {{\varvec{\uplambda}}\left[ {\begin{array}{*{20}c} {v_{x} } \\ {v_{y} } \\ {v_{z} } \\ {\omega_{x} } \\ {\omega_{y} } \\ {\omega_{z} } \\ \end{array} } \right]} \right\} \\ & \quad - \left[ {\begin{array}{*{20}c} 0 &\quad 0 &\quad 0 &\quad 0 &\quad 0 &\quad 0 \\ 0 &\quad 0 &\quad 0 &\quad 0 &\quad 0 &\quad 0 \\ 0 &\quad 0 &\quad 0 &\quad 0 &\quad 0 &\quad 0 \\ 0 &\quad { - v_{z} } &\quad {v_{y} } &\quad 0 &\quad 0 &\quad 0 \\ {v_{z} } &\quad 0 &\quad { - v_{x} } &\quad 0 &\quad 0 &\quad 0 \\ { - v_{y} } &\quad {v_{x} } &\quad 0 &\quad 0 &\quad 0 &\quad 0 \\ \end{array} } \right]\left\{ {{\varvec{\uplambda}}\left[ {\begin{array}{*{20}c} {v_{x} } \\ {v_{y} } \\ {v_{z} } \\ {\omega_{x} } \\ {\omega_{y} } \\ {\omega_{z} } \\ \end{array} } \right]} \right\} \\ \end{aligned}$$

By only considering the vertical plane, Eq. (10) can be simplified as follows:

11 $$\begin{aligned} \left[ {\begin{array}{*{20}c} {F_{ix} } \\ {F_{iy} } \\ {M_{iz} } \\ \end{array} } \right] & = - {\varvec{\uplambda}}\left[ {\begin{array}{*{20}c} {{\text{d}}v_{x} /{\text{d}}t} \\ {{\text{d}}v_{y} /{\text{d}}t} \\ {{\text{d}}\omega_{z} /{\text{d}}t} \\ \end{array} } \right] - {\dot{\mathbf{\lambda }}}\left[ {\begin{array}{*{20}c} {v_{x} } \\ {v_{y} } \\ {\omega_{z} } \\ \end{array} } \right] \\ & \quad - \left[ {\begin{array}{*{20}c} 0 &\quad { - \omega_{z} } &\quad 0 \\ {\omega_{z} } &\quad 0 &\quad 0 \\ 0 &\quad 0 &\quad 0 \\ \end{array} } \right]\left\{ {{\varvec{\uplambda}}\left[ {\begin{array}{*{20}c} {v_{x} } \\ {v_{y} } \\ {\omega_{z} } \\ \end{array} } \right]} \right\} \\ & \quad - \left[ {\begin{array}{*{20}c} 0 &\quad 0 &\quad 0 \\ 0 &\quad 0 &\quad 0 \\ { - v_{y} } &\quad {v_{x} } &\quad 0 \\ \end{array} } \right]\left\{ {{\varvec{\uplambda}}\left[ {\begin{array}{*{20}c} {v_{x} } \\ {v_{y} } \\ {\omega_{z} } \\ \end{array} } \right]} \right\} \\ \end{aligned}$$

In which, $${\varvec{\uplambda}} = \left[ {\begin{array}{*{20}c} {\lambda_{11} } &\quad {\lambda_{12} } &\quad {\lambda_{16} } \\ {\lambda_{21} } &\quad {\lambda_{22} } &\quad {\lambda_{26} } \\ {\lambda_{61} } &\quad {\lambda_{62} } &\quad {\lambda_{66} } \\ \end{array} } \right]$$.

Given the symmetrical characteristic of the vehicle, *λ*_12_ = *λ*_16_ = *λ*_21_ = *λ*_61_ = 0 and *λ*_26_ = *λ*_62_ can be obtained. Then, Eq. (11) can be simplified as:

12 $$\left\{ {\begin{array}{*{20}l} {F_{ix} = - \lambda_{11} \dot{v}_{x} - \dot{\lambda }_{11} v_{x} + \omega_{z} \left( {\lambda_{22} v_{y} + \lambda_{26} \omega_{z} } \right)} \hfill \\ {F_{iy} = - \lambda_{22} \dot{v}_{y} - \dot{\lambda }_{22} v_{y} - \lambda_{26} \dot{\omega }_{z} - \dot{\lambda }_{26} \omega_{z} - \omega_{z} \lambda_{11} v_{x} } \hfill \\ {M_{iz} = - \lambda_{62} \dot{v}_{y} - \dot{\lambda }_{62} v_{y} - \lambda_{66} \dot{\omega }_{z} - \dot{\lambda }_{66} \omega_{z} + v_{y} \lambda_{11} v_{x} - v_{x} \left( {\lambda_{22} v_{y} + \lambda_{26} \omega_{z} } \right)} \hfill \\ \end{array} } \right.$$

where *F*_*ix*_ and *F*_*iy*_ are the ideal fluid force on the vehicle at X and Y directions, respectively, *M*_*iz*_ is the ideal fluid torque of the vehicle at Z direction; *v*_*x*_ and *v*_*y*_ are the vehicle’s velocity component at X and Y directions, respectively, *ω*_*z*_is the angular velocity of the vehicle at Z direction, and *λ* is a term of the added mass. When the vehicle enters water, the diving volume and added mass increase; therefore, the profile analysis method is adopted to calculate the added mass (Лoгвинooвч 1969).

13 $$\left\{ {\begin{array}{*{20}l} {\lambda_{22} = \pi \rho_{0} \int_{{L_{in} }}^{{}} {R^{2} (x)dx} } \hfill \\ {\lambda_{26} = \pi \rho_{0} \int_{{L_{in} }}^{{}} {R^{2} (x)xdx} } \hfill \\ {\lambda_{66} = \pi \rho_{0} \int_{{L_{in} }}^{{}} {R^{2} (x)x^{2} dx} } \hfill \\ \end{array} } \right.$$

The added mass *λ*_11_ of slender body is extremely small (Liao et al. 2012), therefore *λ*_11_ is set to zero. At the same time, $\dot{\lambda }_{11}$ equals zero. By differentiating both sides of Eq. (13), the change rate of added mass can be obtained as follows:

14 $$\left\{ {\begin{array}{*{20}l} {d\lambda_{22} = \pi \rho_{0} R^{2} dx_{a} } \hfill \\ {d\lambda_{26} = \pi \rho_{0} L_{in} R^{2} dx_{a} } \hfill \\ {d\lambda_{66} = \pi \rho_{0} L_{in}^{2} R^{2} dx_{a} } \hfill \\ \end{array} } \right.$$

In Eq. (14), *dx*_*a*_ is the change rate of the vehicle’s front part that entered the free surface of water, and *dx*_*a*_ = *v*_*x*_ *dt*. *R* is the radius of the vehicle, as shown in Eq. (1).

➁ Viscous fluid force *F*_*μ*_

Experiment and theoretical analysis results indicate that the motion drag coefficient of underwater vehicle is closely related to the velocity, Reynold number, and attack angle (Michael and Jerry 1976). The effect of the varying Reynold number can be ignored here because the vehicle’s speed is assumed low and does not vary in a large scale.

The computational fluid dynamics (CFD) method can be used to calculate the drag *F*_*μx*_, lift *F*_*μy*_, moment of force *M*_*μz*_ in a given speed, and the attack angle when the vehicle is full immersion. In this study, ANSYS CFX 14.0 is used to calculate the drag *F*_*μx*_, lift *F*_*μy*_, and moment of force *M*_*μz*_.

**Fig. 3:**
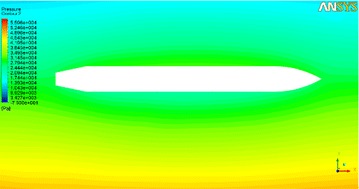
The pressure nephogram of the vehicle

As seen in Fig. 3, the pressure nephogram of the vehicle when the fluid speed is 20 m/s and the attack angle is zero. The calculation step is set to 0.01 ms, and the end time is 0.01 s. That is, the result is derived by calculating 1000 steps. Then, the drag *F*_*μx*_, lift *F*_*μy*_, and moment of force *M*_*μz*_ can be obtained.

The viscous fluid dynamic coefficient *C*_*d*_, *C*_*l*_, and *m*_*z*_ can be calculated with Eq. (15).

15 $$\left\{ {\begin{array}{*{20}l} {C_{d} = \frac{{F_{\mu x} }}{{0.5\rho_{0} v^{2} }}} \hfill \\ {C_{l} = \frac{{F_{\mu y} }}{{0.5\rho_{0} v^{2} }}} \hfill \\ {m_{z} = \frac{{M_{\mu z} }}{{0.5\rho_{0} v^{2} }}} \hfill \\ \end{array} } \right.$$

**Fig. 4:**
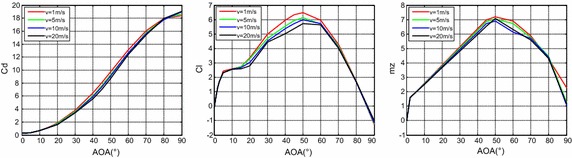
The dynamic coefficient under diffident speed and attack angle

The fluid dynamic coefficient under different speeds and attack angles can be calculated through the same method. However, a huge workload is required to calculate the coefficient under every speed and attack angle, which is time consuming. The table lookup interpolation method is adopted to reduce the workload. Considering that the fluid dynamic coefficient is minimally influenced by speed, the speeds 1, 5, 10, 20 m/s and the attack angles 0°, 10°, 20°, …, 90°, are chosen. Figure 4 displays the result.

From Fig. 4, it can be seen that, the drag coefficient *C*_*d*_ increased with the increase of the attack angle, while the lift coefficient *C*_*l*_ and the moment coefficient *m*_*z*_ increase at first when *α* < 50°, then decrease when *α* > 50°. The changing law of *C*_*l*_ and *m*_*z*_ resulted from the shape of the vehicle and the turbulent flow theory.

According to the calculation by CFD, a one-to-one correspondence exists between the viscous fluid dynamic coefficient and the changing relationship of the attack angle and speed. Therefore, three two-dimensional interpolation tables of the viscous fluid dynamic coefficient can be established, in which the line represents the speed and the row represents the attack angle. Based on the current speed and angle, the viscous fluid dynamic coefficient is calculated by interpolation. Then, the viscous fluid force during water entry can be calculated. The viscous fluid dynamic coefficient can be calculated by the equation below.

16 $$\left\{ {\begin{array}{*{20}l} {C_{d} = f_{1} (v,\alpha )} \hfill \\ {C_{l} = f_{2} (v,\alpha )} \hfill \\ {m_{z} = f_{3} (v,\alpha )} \hfill \\ \end{array} } \right.$$

The fluid dynamic coefficient calculated is under the condition that the vehicle is in full immersion. In practice, the vehicle is partly immersed when crossing the water surface. Given that the fluid dynamic coefficient increases as the wetted area increases, the relationship between them can be assumed linear. The viscous fluid force during water entry can be obtained by:

17 $$\left\{ {\begin{array}{*{20}l} {F_{\mu x} = - \frac{1}{{2S_{0} }}C_{d} \rho_{0} v^{2} S} \hfill \\ {F_{\mu y} = \frac{1}{{2S_{0} }}C_{l} \rho_{0} v^{2} S} \hfill \\ {M_{\mu z} = F_{\mu y} \cdot \frac{1}{2}x_{a} } \hfill \\ {S_{0} = 2\pi \int_{0}^{L} {R(x)dx} } \hfill \\ {S = 2\pi \int_{0}^{{x_{a} }} {\int_{0}^{L} {R(x)dx} } } \hfill \\ {v = \sqrt {v_{x}^{2} + v_{y}^{2} } } \hfill \\ \end{array} } \right.$$

In the equation, *S*_0_ is the superficial area of the vehicle, and *S* is the wetted area of the vehicle. After the vehicle completely immerses into water, the pitch moment caused by viscous fluid influence is:

18 $$M_{\mu z} = \frac{1}{2}m_{z} \rho_{0} v^{2}$$

### Dynamic model

According to the force analysis above, a dynamic model of water entry is established below (Xu et al. 2015).

19 $$\left\{ {\begin{array}{*{20}l} {F_{ix} + F_{\mu x} + (B - W)\sin \theta = m\left( {dv_{x} /dt - v_{y} \omega_{z} } \right)} \hfill \\ {F_{iy} + F_{\mu y} + (B - W)\cos \theta = m\left( {dv_{y} /dt + v_{x} \omega_{z} } \right)} \hfill \\ {M_{iz} + M_{\mu z} + Bx_{b} \cos \theta = J \cdot d\omega_{z} /dt} \hfill \\ \end{array} } \right.$$

In the equation, *J* is the moment of inertia of the vehicle, which can be obtained by the moment of inertia theorem of the disc shape and parallel axis formula.

20 $$J = \pi \rho_{0} \int_{0}^{L} {R^{2} (x)\left[ {1/4R^{2} (x) + \left( {x - x_{0} } \right)^{2} } \right]} dx$$

The differential equation set, which can describe the water-entry process of vehicle consists of Eqs. (1)–(20). Based on the given initial value condition, the equation set can be employed with the classic four-order Runge–Kutta iteration algorithm. The initial value condition includes the initial speed (*v*_*x*0_, *v*_*y*0_), initial water-entry angle *θ*_0_, initial rotational angular speed *ω*_*z*0_, and initial distance *x*_*a*0_, which is between the head peak of the vehicle and water surface along the axial direction.

## Experiment and data processing

### Experiment model

**Fig. 5:**
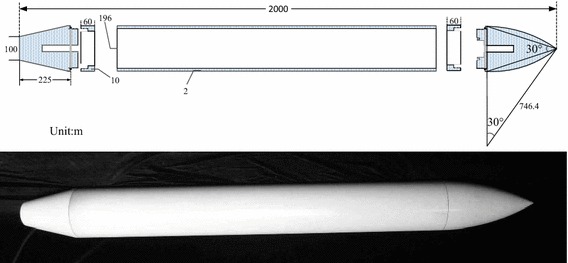
Size and physical diagram of experiment model

To verify the accuracy and suitability of the dynamic model, a water-entry experiment was performed on the model. Figure 5 depicts the size and physical diagram of experiment model. The model was composed of head, stern, and body. The head and stern were made of aluminum. The body was made of standard steel tube. The head and stern were assembled to the body with screw thread. The waterproofing gasket was used to guarantee the air tightness. The cylindrical quirk in the head and stern reduced the weight of the experiment model. An acceleration sensor was placed in the cylindrical quirk in the back head. The model weighed 65.1 kg, the volume was 0.052 m^2^, the mean density was 1.252 kg/m^3^, and the rotary inertia was 33.56 kg m^2^. In addition, the barycenter is nearer to the head and 1.08 m to the stern.

### Experiment process

On-shore and underwater high-speed cameras were used during the experiment. The on-shore high-speed camera filmed the trajectory from the launch to the water entry. The underwater high speed camera filmed the trajectory of the under-water attitude. An acceleration sensor was fixed on the model head to record the acceleration of the entire process. The experiment model was launched from the second floor to the pool by a high-pressure launch set. Different water-entry experiments can be realized by changing the experiment conditions, such as angle of launcher support and pressure.

### Experiment data processing

1. Data processing of on-shore high speed camera

**Fig. 6:**
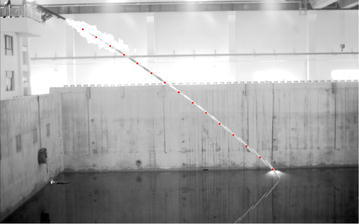
Air trajectory of vehicle model

The on-shore high-speed camera was set to 1000 fps to film the air trajectory of the experiment model. The video was then sampled every 50 fps (Δ*t* = 0.05 s). The head coordinate (*X*_*head*_, *Y*_*head*_) and stern coordinate (*X*_*tail*_, *Y*_*tail*_) of the launcher point were obtained. The linear logistic algorithm was adopted to estimate the dynamic state of the experiment model. The detail procedures were as follows:

➀ The center-of-gravity position was 1.08 m from the stern throughout the experiment. The coordinates of the center-of-gravity position and slope angle were derived from Eqs. (21) and (22). Figure 6 depicts the air trajectory of the model. The red dots denote the center-of-gravity position.

  21 $$\left( {X_{cg} ,Y_{cg} } \right) = \frac{1.08}{2}\left( {X_{head} ,Y_{head} } \right) + \frac{0.92}{2}\left( {X_{tail} ,Y_{tail} } \right)$$
  22 $$\theta = \text{arc} \,tan\left( {\frac{{Y_{head} - Y_{tail} }}{{X_{head} - X_{tail} }}} \right)$$
➁ Linear regression algorithm was adopted to calculate the initial state of the air trajectory $(X_{0} ,v_{x0} ,Y_{0} ,v_{y0} ,\theta_{0} ,\omega_{0} )$. The model considered gravity regardless of air resistance. The center-of-gravity position and slope angle were:
  23 $$X_{cg} = X_{0} + v_{x0} t$$
  24 $$Y_{cg} = Y_{0} - v_{y0} t - \frac{1}{2}gt^{2}$$
  25 $$\theta = \theta_{0} + \omega_{0} t$$
➂ At the water-entry moment *T* = *t*_*in*_, horizontal velocity can be denoted as *V*_*x*_*in*_ = *v*_*x*0_, vertical velocity *V*_*y*_*in*_ = *v*_*y*0_ + *gt*_*in*_, and span rate *ω*_*in*_ = *ω*_0_. The coordinate of the center-of-gravity position and slope angle were directly read from the pictures. Then, the dynamic state $(X_{in} ,v_{x\_in} ,Y_{in} ,v_{y\_in} ,\theta_{in} ,\omega_{in} )$ was obtained.

2. Data processing of under-water high speed camera

**Fig. 7:**
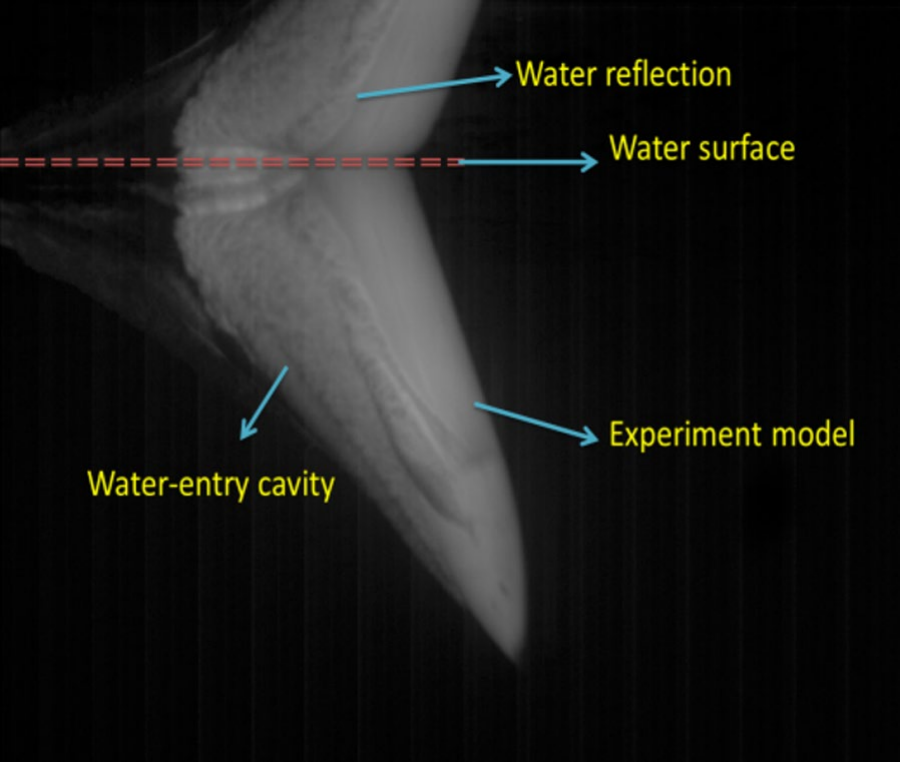
Picture of the model under water at t = 0.04 s

**Fig. 8:**
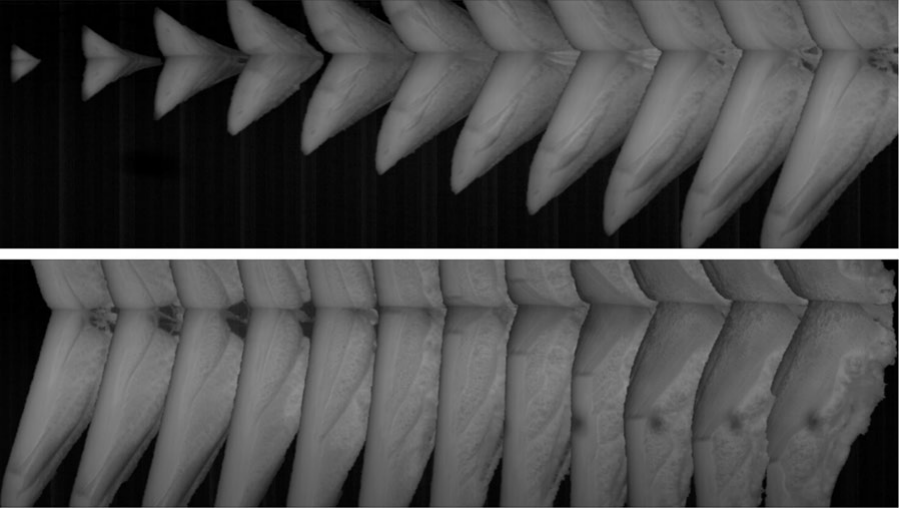
Under-water picture of vehicle model in water-entry process (interval time is 0.005 s)

The underwater high-speed camera filmed the water-entry process. The vision field of underwater high-speed camera was narrow, and the time of motion in the vision field was short. The video was sampled every 5 fps, that is, Δ*t* = 0.005 s, to accurately show the change of dynamic state when the vehicle model entered the water. When the launch angle was 30° and the initial pressure was 0.7 Mpa, the underwater high-speed camera shot the picture at 0.04 s after the vehicle model entered the water. The light area showed the bubbles caused by the water entry. The dark area showed the water. The vehicle model, bubbles, and the inverted reflection were easily distinguished in water, as depicted in Fig. 7. Figure 8 depicts the change of the model’s attitude over time.

**Fig. 9:**
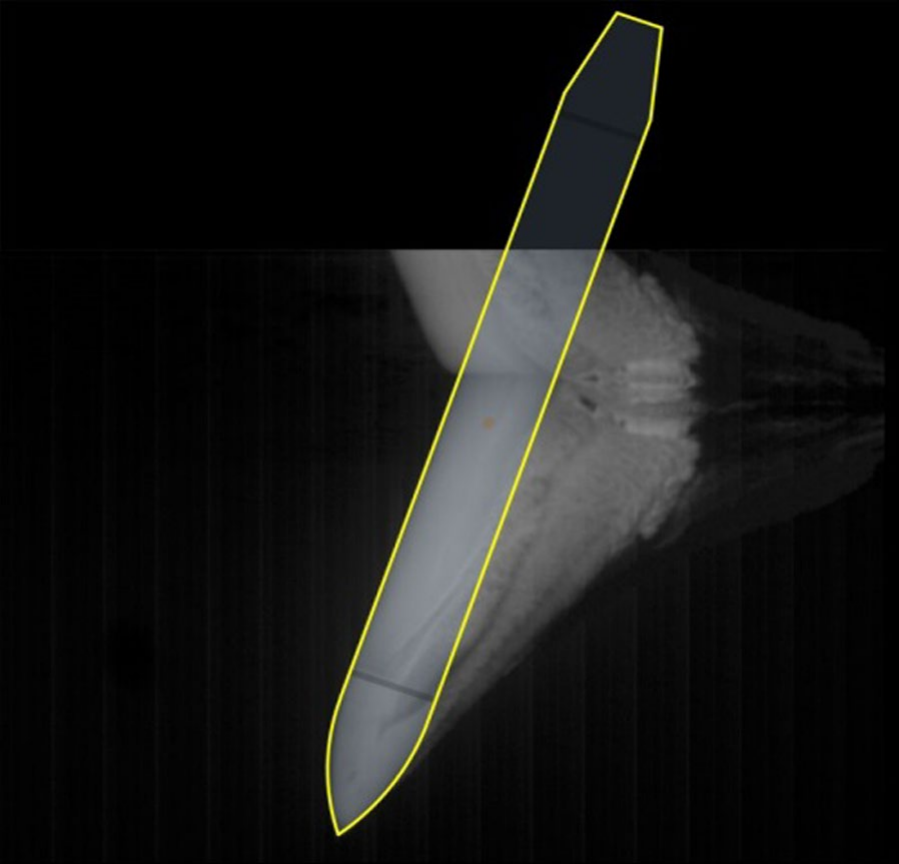
Determination of model outline
➁ The slope angle was acquired through the easily recognizable front profile, as shown in Fig. 10. The first three pictures were neglected because of the limited water-entry time and vague slope angle.

Illustrating the vehicle model in the completely same scene was difficult because the vision of underwater high-speed camera was narrow. To obtain the attitude and center-of-gravity position during water entry, the pictures were processed as follows:

➀ The picture showing the complete vehicle model was adopted as the baseline. The outline of the model in picture was calculated according to the actual proportional size, as depicted in Fig. 9.

**Fig. 10:**
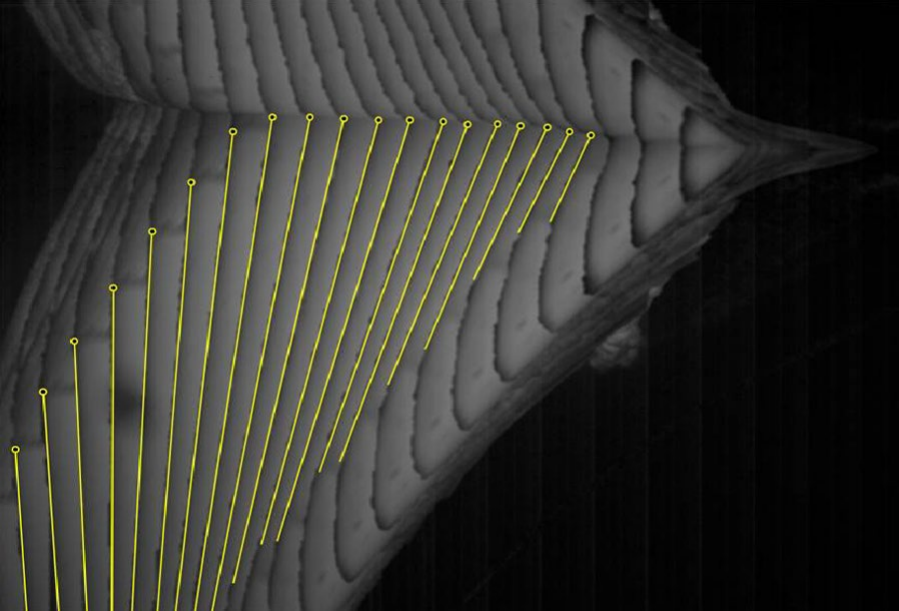
Determination of model slope angle (interval time is 0.005 s)
➂ The position and attitude were obtained according to the outline size and slope angle. Then, the center-of-gravity positions were obtained according to the propositional size. Figure 11 depicts the change of attitude. The yellow line represents the outline, and the red line indicates the water surface.

**Fig. 11:**
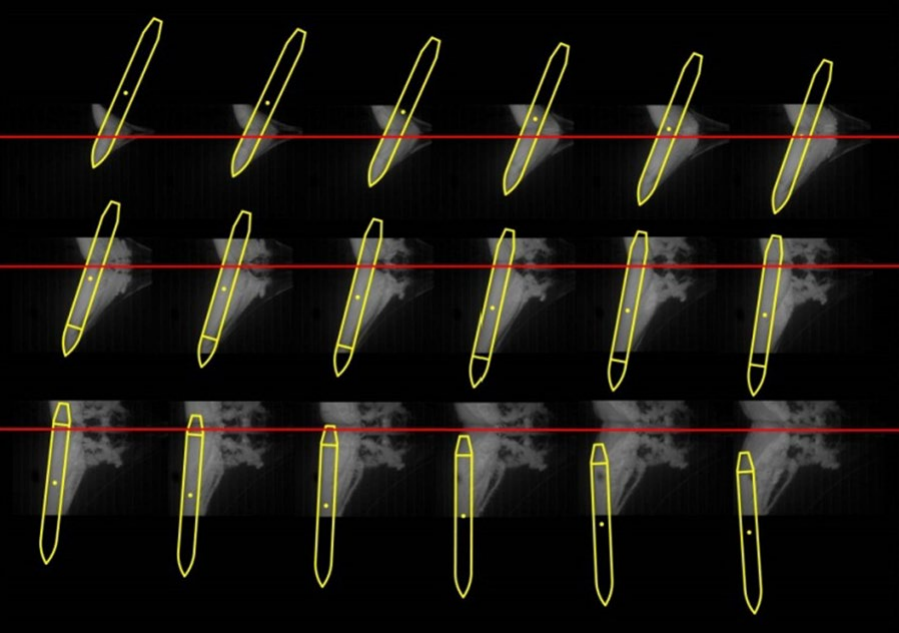
Determination of the model attitude (interval time is 0.005 s)

3. Data processing of acceleration sensor

**Fig. 12:**
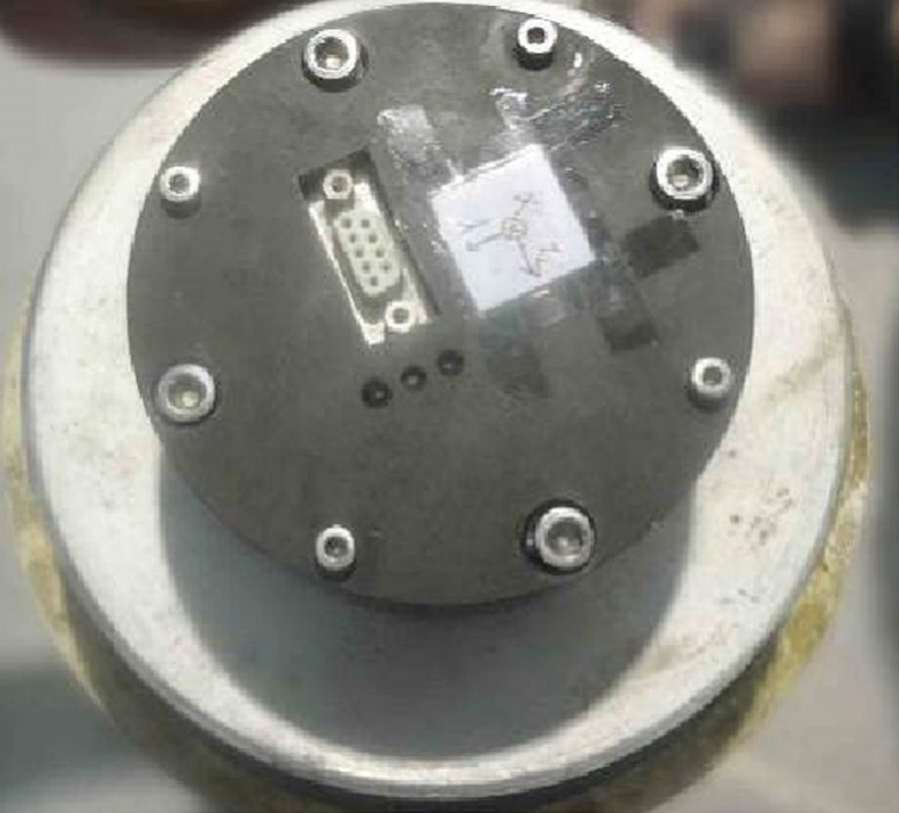
Acceleration sensor fixed to the head of the vehicle

The acceleration sensor, as depicted in Fig. 12, recorded the change of impact loading from the launch to the complete water-entry process. This study investigated the water entry. The entire process only lasted < 0.1 s according to the data of the acceleration sensor. This study investigated the 0.16 s interval after the model entered water. And the X axis points the head of the vehicle, while the Y axis points up in the vertical plane.

## Model verification

To verify the accuracy of the proposed dynamic equation, experiment and simulation were carried out under the same condition, and the results were compared. The water-entry state was not under control because of the restriction of experiment condition. With the pressure of 0.7 Mpa and launch angle of 30°, the initial water-entry state parameters of different launcher angles were obtained using the proposed experiment data processing. The horizontal velocity, vertical velocity, span rate, attack angle, and slope angle at the water-entry moment were derived as 21.7, 19.48 m/s, 25.43°/s, 18.02°, and 59.93°, respectively. Table 1 shows the change of attitude and center-of-gravity position over time in the experiment.

**Table 1:** Change of attitude and center-of-gravity position of the model

Time (s)	0.02	0.025	0.03	0.035	0.04	0.045	0.05	0.055	0.06
Slope angle (°)	63.93	64.96	65.82	68.39	68.75	70.71	71.85	73.72	75.64
X (mm)	1600.0	1493.9	1396.9	1272.2	1180.5	1075.3	983.9	891.6	801.6
Y (mm)	1531.1	1431.9	1341.4	1242.9	1125.1	1020.3	913.8	799.5	689.4
Time (s)	0.065	0.07	0.075	0.08	0.085	0.09	0.095	0.1	0.105
Slope angle (°)	77.57	79.59	81.71	83.72	85.78	87.18	89.75	92.44	93.74
X (mm)	732.1	651.4	588.9	522.3	458.6	394.1	347.7	299.5	247.3
Y (mm)	601.0	522.3	444.5	353.4	256.7	137.6	15.6	−111.0	−209.8

**Fig. 13:**
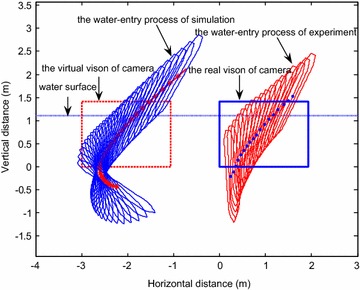
Comparison of the results of simulation and experiment

**Fig. 14:**
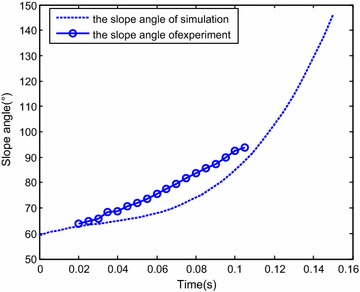
Comparison of the slope angel

**Fig. 15:**
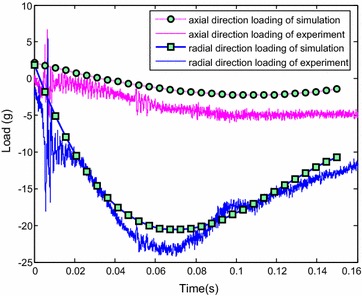
Comparison of change of axial direction

The initial simulation condition was set to the same value as the initial water-entry state parameter in the experiment. The simulation time was set to 0.15 s. Figure 13 displays the results of experiment and simulation. The blue solid box shows the vision field of the underwater camera. The red dotted box is the counterpart in the simulation. Figure 14 shows the comparison of the change of slope angle, and Fig. 15 shows the comparison of the change of axial direction (X axis) and radial direction (Y axis) loading. In Fig. 15, when the vehicle entered the water, the radial direction loading of the vehicle plummeted to −25 g at around 0.07 s. It indicates that when the vehicle entered the water, the radial direction would withstand a big overload at around 0.07 s. Figures 13, 14 and 15 reveal that the simulation was consistent with the experiment, thus the proposed dynamic equations were reasonable. Nevertheless, some errors were encountered. The main causes were as follows:

1. The simulation neglected the effects of perturbation of the water surface, water-entry cavity, and splashing.
2. The model was treated as rigid body in simulation, which was inconsistent with reality.
3. The viscous hydrodynamic coefficients were calculated by interpolation, which caused the errors.
4. Errors also existed during data acquisition and processing.

In addition, there are some limitations of the dynamic model: (1) the model can be applied for low-velocity problem only; (2) the model can be applied to axisymmetric body or symmetric flow only; (3) usually, the added mass method is only stable when the body mass is larger than the added liquid mass due to the moving body.

From the results of experiment and simulation, overturning can easily happen. The overturn phenomenon is strongly connected with the initial water-entry angle of attack and rotational angular velocity.

## Model simulation

Using the proposed dynamical model, other simulations were run to reveal the relationship among the overturn, and angle of attack, and rotational angular velocity.

### Influence of initial angle of attack

**Fig. 16:**
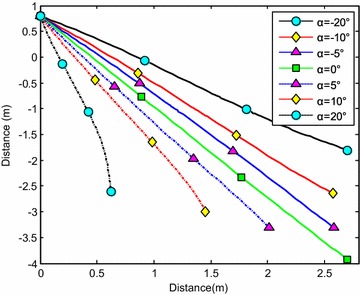
Trajectories of center-of-gravity position

**Fig. 17:**
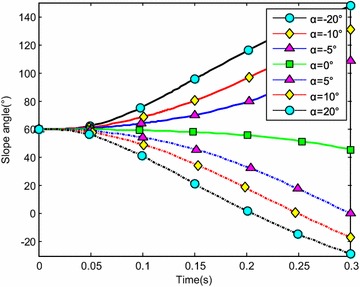
The change of slope angles

**Fig. 18:**
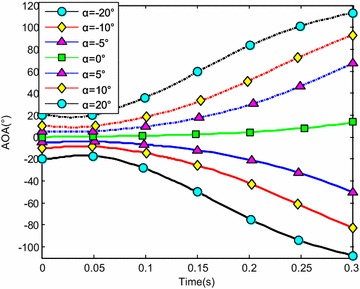
The change of angle of attack

**Fig. 19:**
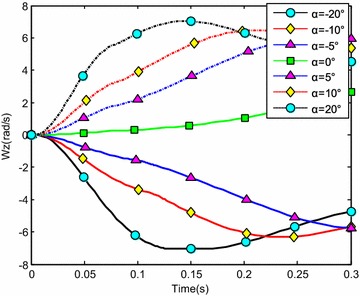
The change of rotational angular velocity

The water-entry velocity was reset to 20 m/s, water-entry to 60°, and rotational angular to 0. The initial water-entry angle of attack was set to 0°, ±5°, ±10°, and ±20°, considering the structure tolerance of the water-entry impact force (Yang et al. 2001). The simulation results are shown in Figs. 16, 17, 18, and 19.

The results showed that when *α* < 0°, the increase of the absolute angle of attack value decreased water-entry depth, horizontal displacement, and slope angle. It also accelerated the increase of negative rotational angular velocity, negative angle of attack, and slope angle. The vehicle rotated downwards and even overturned. When *α* ≥ 0°, the decrease of initial angle of attack value decreased water-entry depth and horizontal displacement and accelerated the decrease of slope angle. It accelerated the increase of positive rotational angular velocity and positive angle of attack. The vehicle rotated downwards and even overturned. The decrease of initial water-entry angle of attack reduced the change of slope angle and angle of attack during water entry. The trajectory was more stable, thus impeding the overturn phenomenon.

When the vehicle reached the water surface, the fluid torque influenced the motion of vehicle and increased with high initial angle of attack. As the vehicle entered water, the buoyancy and buoyancy center changed. The misalignment between center-of-gravity position and buoyant center resulted to another torque. Both torques were affected simultaneously. When *α* < 0°, the initial angle of attack and fluid torque were both negative. The vehicle began to rotate downwards. The increase of slope angle caused the negative increase of the angle of attack and rotational angular velocity. Both torques accelerated the change of angle of attack and slope angle when the vehicle entered the water completely. The overturn occurred downwards when the slope angle exceeded the threshold. When *α* ≥ 0°, the initial angle of attack and fluid torque were both positive. The vehicle began to rotate upwards. The decrease of slope angle caused the positive increase of the angle of attack and rotational angular velocity. Both torques accelerated the change of angle of attack and slope angle when the vehicle entered the water completely. The overturn occurred upwards when the slope angle exceeded the threshold. Choosing the proper water-entry angle of attack is important to avoid the overturn phenomenon.

### Influence of initial rotational angular velocity

**Fig. 20:**
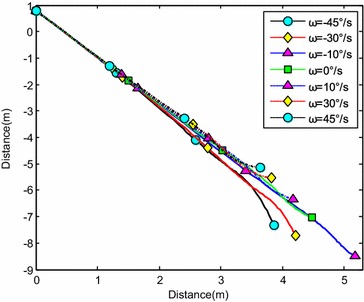
Trajectories of center-of-gravity position

**Fig. 21:**
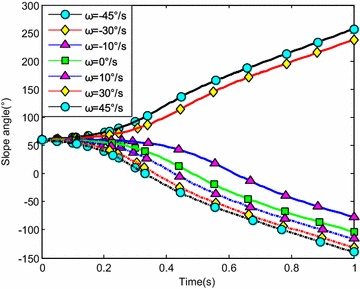
The change of slope angles

**Fig. 22:**
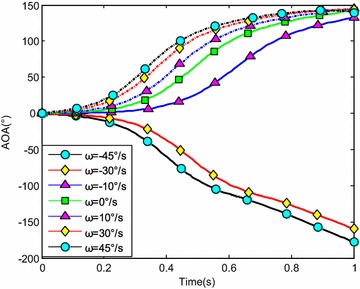
The change of angle of attack

**Fig. 23:**
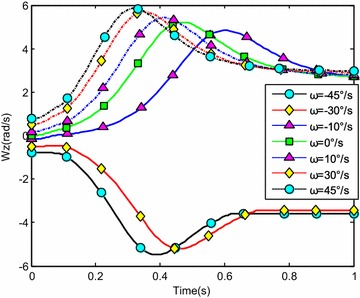
The change of rotational angular velocity

The water-entry velocity was reset to 20 m/s, water-entry to 60°, and initial water-entry angle of attack to 0°. The initial water-entry rotational angular velocity was set to 0°/s, ±10°/s, ±30°/s, and ±45°/s. The simulation results are shown in Figs. 20, 21, 22, and 23.

The results showed that initial rotational angular velocity affected the water-entry process. The effects of the two torques were the same as those mentioned above. When *ω* ≥ −10°/s, the increase of initial rotational angular velocity accelerated the decrease of water-entry depth, horizontal displacement, and slope angle. It also accelerated the increase of positive rotational angular velocity and angle of attack. The vehicle rotated upwards and even overturned. When *ω* ≤ −30°/s, the decrease of initial rotational angular velocity accelerated the decrease of water-entry depth, horizontal displacement, rotational angular velocity, and angle of attack. It also accelerated the increase of slope angle. The vehicle rotated downwards and even overturned. Choosing the proper water-entry initial rotational angular velocity is crucial to prevent the overturn phenomenon.

### Water-entry control

Water entry was difficult to control because the process was transient and complex. The initial angle of attack and initial rotational angular velocity influenced the water-entry trajectory. The vehicle was more stable with a smaller angle of attack after entering the water completely. Vehicle attitude should be controlled before water entry to guarantee stability after entering the water completely.

Attitude control mechanism could adjust the initial rotational angular velocity to achieve the navigation with zero-attack after entering the water completely. The dynamic model proposed in “Establishment of dynamical model” section is a multivariate differential equation set. Finding an analytical solution is difficult, and, thus, this problem can be solved by converting it to constraint optimization problem.

26 $$\begin{aligned} & \hbox{min} \left| \alpha \right| = U\left( {\omega_{{z{-}begin}} } \right) \\ & \quad {\text{s}}.{\text{t}}.\,\left| {\omega_{{z{-}begin}} } \right| < \pi /T \\ \end{aligned}$$

where function *U*(*ω*) is the proposed dynamic model in “Establishment of dynamical model” section, *ω*_*z*-*begin*_ is the controllable initial rotational angular velocity, and *α* is the angle of attack. When an initial rotational angular velocity *ω*_*z*-*begin*_ was given, we can use the dynamic model proposed in “Establishment of dynamical model” section to calculate the attack angle *α*. In the constraint condition, *T* is the total time to complete the water-entry process. The condition can be avoided by deriving a substantial calculated *ω*_*z*-*begin*_, which may cause a larger *π* angle variation of the inclination angle before the vehicle completely enters the water.

In Eq. (26), *ω*_*z*-*begin*_ is the input variable of *U*(*ω*), which allows |*α*| to be minimum. It is also a unimodal function according to the real vehicle motion. The golden section algorithm (Chen 2005) was adopted to search for the optimal value.

- *Step 1* Set area [*a*_1_, *b*_1_] = [−*π*/*T*, *π*/*T*], *k* = 1 and *T* = 0.2; the accuracy *W* = 10^−4^ rad s. Calculate *λ*_1_ = *a*_1_ + 0.382(*b*_1_ − *a*_1_) and *μ*_1_ = *a*_1_ + 0.618(*b*_1_ − *a*_1_), and use the proposed dynamic model in “Establishment of dynamical model” section calculate *U*(*λ*_1_) and *U*(*μ*_1_).
- *Step 2* If *b*_*k*_ − *a*_*k*_ < *W*, stop. Else, if *U*(*λ*_1_) > *U*(*μ*_1_), go to step 3; if *U*(*λ*_1_) ≤ *U*(*μ*_1_), go to step 4.
- *Step 3* Set *a*_*k*+1_ = *λ*_*k*_, *b*_*k*+1_ = *b*_*k*_, *λ*_*k*+1_ = *μ*_*k*_, *μ*_*k*+1_ = *a*_*k*+1_ + 0.618(*b*_*k*+1_ − *a*_*k*+1_), calculate *U*(*μ*_*k*+1_), go to step 5.
- *Step 4* Set *a*_*k*+1_ = *a*_*k*_, *b*_*k*+1_ = *μ*_*k*_, *μ*_*k*+1_ = *λ*_*k*_, *λ*_*k*+1_ = *a*_*k*+1_ + 0.382(*b*_*k*+1_ − *a*_*k*+1_), calculate *U*(*λ*_*k*+1_), go to step 5.
- *Step 5* *k* = *k* + 1, go to step 2.

**Fig. 24:**
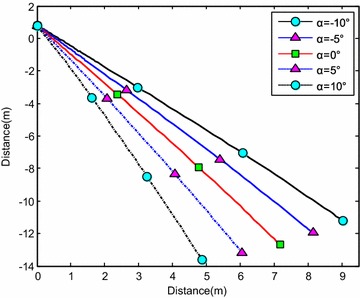
Trajectories of center-of-gravity position

**Fig. 25:**
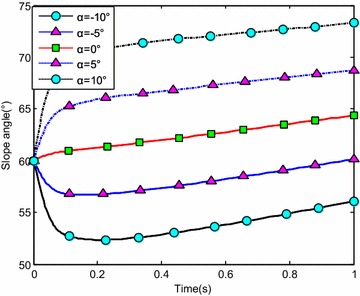
The change of slope angles

**Fig. 26:**
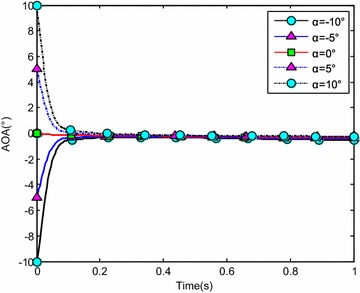
The change of angle of attack

**Fig. 27:**
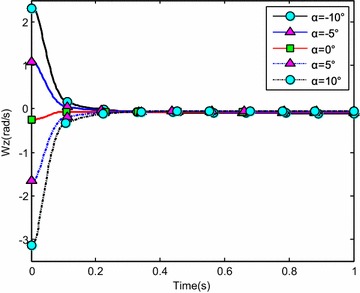
The change of rotational angular velocity

Table 2 shows the optimal rotational angular velocity when water-entry velocity was 20 m/s, slope angle was 60°, and angle of attack were 0°, ±5°, and ±10°. The results are shown in Figs. 24, 25, 26, and 27.

**Table 2:** Optimization results of water-entry rotational angular velocity

Water-entry angle of attack (°)	−10	−5	0	5	10
Optimal rotational angular velocity (rad/s)	2.3144	1.0845	−0.2468	−1.6628	−3.1377

Simulation results showed that, with the optimal initial rotational angular velocity, the slope angle changed slightly. After entering the water, the angle of attack and rotational angular velocity approached zero quickly. The trajectory was stable and straight; thus, the overturn phenomenon was avoided, which was beneficial for the subsequent underwater control.

## Conclusions

This study modelled the water entry of submersible aircraft to investigate the water-entry attitude and overturn phenomenon. Simulation and experiment were carried out to verify the accuracy of the proposed model. The following conclusions were drawn:

1. The initial angle of attack and initial rotational angular velocity influenced the water-entry attitude and overturn phenomenon.
2. The trajectory was stable with a decreased angle of attack after entering the water.
3. An optimization function was established to solve the optimal initial rotational angular velocity, thus increasing the water-entry attitude and underwater navigation stability of vehicle.

## Abbreviation

CFD: computational fluid dynamics
